# Supplementary material for: Quasi-exact solutions for guided modes in two-dimensional materials with tilted Dirac cones
Source: Sci Rep. 2022 May 10;12:7688. doi: 10.1038/s41598-022-11742-3 (PMC9091279; doi:10.1038/s41598-022-11742-3)
Supplement: Supplementary file 1 — Supplementary Information. [file 41598_2022_11742_MOESM1_ESM.pdf]

Supplementary information: Quasi-exact solutions for guided modes in two-dimensional materials with tilted Dirac cones

R. A. Ng[1], A. Wild[2], M. E. Portnoi[2,3,+], and R. R. Hartmann[1,*]

[1]Physics Department, De La Salle University, 2401 Taft Avenue, 0922 Manila, Philippines
[2]Physics and Astronomy, University of Exeter, Stocker Road, Exeter EX4 4QL, United Kingdom
[3]ITMO University, St. Petersburg 197101, Russia
[*]Richard.hartmann@dlsu.edu.ph
[+]M.E.Portnoi@exeter.ac.uk

ABSTRACT

In this Supplementary Information we present a full description of the transfer matrix method used to calculate the band structure of guiding potentials in 2D Dirac materials with tilted Dirac cones.

Numerical Model of a Waveguide in a 2D Dirac Material with Tilted Dirac Cones

In order to confirm the results of the main text, we discretize smooth waveguides and numerically calculate their band structure using the transfer matrix method. We begin by determining the wavefunction in a single 1D square well for a Dirac material with tilted cones, as an extension to the graphene case[1]. An arbitrary number of square wells are placed one after another to approximate the smooth waveguides discussed in the main text. For the system of sequential wells we set up a transfer matrix which links the wavefunction components in the leftmost and rightmost well. Bound states yielding the band structure are found numerically by placing boundary conditions on the transfer matrix. The boundary conditions ensure the wavefunction has plane-wave solutions inside the waveguide and decaying solutions at the edges of the system.

Solution to the Dirac Equation with Tilted Dirac Cones in a Square Well

The Dirac equation for electrons in a 2D semimetal with tilted Dirac cones subjected to the potential $U(x,y)$ has the form

$$v_x\big(st\sigma_0\hat{p}_y + sT\sigma_y\hat{p}_y + \sigma_x\hat{p}_x\big)\Psi(x,y) = \big(\varepsilon - U(x,y)\big)\Psi(x,y), \tag{S1}$$

with energy ε, momentum $\hat{p}_i$ where $i = x,y$, Fermi velocity v_x, tilt parameter t, anisotropy parameter T and valley index $s = \pm 1$. The identity matrix is σ_0, the Pauli matrices are σ_x and σ_y, and the spinor wavefunction is $\Psi(x,y) = (\phi^{\mathrm{A}}(x,y), \phi^{\mathrm{B}}(x,y))^\intercal$ which is written in the basis of the Bloch sums of the Dirac material. We wish to find the wavefunction of the jth well where $j = 1,2,...N$. Each well has finite depth U_j with arbitrary width along the x'-axis ($x'_{j-1} \leq x' \leq x'_j$) and is invariant along the perpendicular y'-axis (see Fig. 1 of the main text). Next we perform a coordinate transformation on the momentum operators $\hat{p}_x = \hat{p}_{x'}\cos\theta - \hat{p}_{y'}\sin\theta$ and $\hat{p}_y = \hat{p}_{x'}\sin\theta + \hat{p}_{y'}\cos\theta$ before inserting their definition $\hat{p}_{i'} = -i\hbar\partial_{i'}$, where $i' = x', y'$. Due to the invariance along the y'-axis we can insert a plane-wave solution along this axis $\Psi_j(x',y') = \Psi_j(x')e^{ik_{y'}y'}$. It is at this point that we introduce an arbitrary length scale L allowing us to define the dimensionless parameters $\xi = x'/L$, $V_j = U_jL/\hbar v_x$, $E = \varepsilon L/\hbar v_x$ and $\Delta = k_{y'}L$. Let the wavefunction in the jth well be of the form $\Psi_j(\xi) = (\phi_j^{\mathrm{A}}, \phi_j^{\mathrm{B}})^\intercal e^{ib_j\xi}$, where b_j is in general a complex number. All preceding steps yield the eigenvalue problem

$$\begin{pmatrix} st\big(b_j\sin\theta + \Delta\cos\theta\big) - \big(E - V_j\big) & b_j\cos\theta - \Delta\sin\theta - isT\big(b_j\sin\theta + \Delta\cos\theta\big) \\ b_j\cos\theta - \Delta\sin(\theta) + isT\big(b_j\sin\theta + \Delta\cos\theta\big) & st\big(b_j\sin\theta + \Delta\cos\theta\big) - \big(E - V_j\big) \end{pmatrix} \begin{pmatrix} \phi_{j,\mathrm{A}} \\ \phi_{j,\mathrm{B}} \end{pmatrix} = \mathbf{0}, \tag{S2}$$

where $\mathbf{0}$ is the null vector. We define the above eigenvalue problem as $[\mathscr{H}_j - \sigma_0(E - V_j)](\phi_j^{\mathrm{A}}, \phi_j^{\mathrm{B}})^\intercal = \mathbf{0}$, where for non-trivial solutions we must ensure $|\mathscr{H}_j - \sigma_0(E - V_j)| = 0$. This step yields two solutions ($\pm$) for the complex parameter

$$b_{j,\pm} = \frac{\big[st(V_j - E) + (1 + t^2 - T^2)\Delta\cos\theta\big]\sin\theta \pm \sqrt{(t^2 - T^2)\Delta^2 + 2st\Delta(V_j - E)\cos\theta + l^2(V_j - E)^2}}{l^2 - t^2\sin^2\theta}, \tag{S3}$$

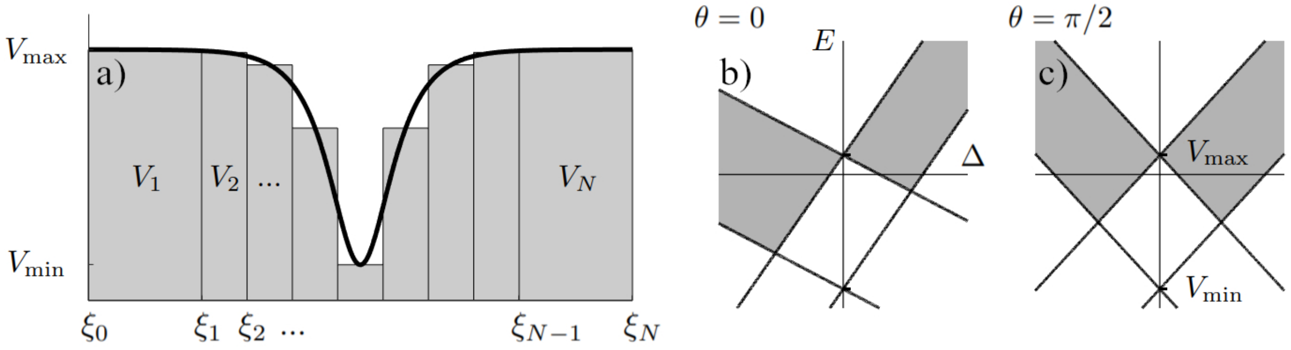

Figure S1. (a) Schematic of the waveguide $V(\xi) \propto -1/\cosh(\xi)$ approximated by N square wells. The depth of the jth well is V_j and the leftmost and rightmost wells (V_1 and V_N) are arbitrarily wide as it is assumed $\xi_0 \to -\infty$ and $\xi_N \to \infty$. The waveguide has a depth of V_{min} and will terminate at the edges with a potential of V_{max}. Panels (b) and (c) represent the regions of wavevectors and energies where the waveguide has bound states in valley $s = 1$, for waveguide orientations $\theta = 0$ and $\pi/2$ respectively, when $|T| \neq 1$ and $0 < |t| < |T|$. The results for the second valley $s = -1$ can be found from the $s = 1$ case by replacing Δ with $-\Delta$.

where $l = \sqrt{1-(1-T^2)\sin^2\theta}$. The first term of Eq. (S3) is always real whilst the second term can be either real or imaginary depending on the parameters. We shall now introduce the subscript $\pm$ corresponding to the two roots of $b_{j,\pm}$. Inserting these solutions back into Eq. (S2) allows us to define $\phi^{\text{B}}_{j,\pm} = \Lambda_{j,\pm}\phi^{\text{A}}_{j,\pm}$ where

$$\Lambda_{j,\pm} = \frac{-st\left(b_{j,\pm}\sin\theta + \Delta\cos\theta\right) + (E - V_j)}{b_{j,\pm}\cos\theta - \Delta\sin\theta - isT\left(b_{j,\pm}\sin\theta + \Delta\cos\theta\right)}. \tag{S4}$$

The wavefunction in the region $\xi_{j-1} \leq \xi \leq \xi_j$, where $\xi_j = x'_j/L$ can be written as a linear combination of the forward and backward propagating plane-wave solutions:

$$\Psi_j(\xi) = C_j\left[\phi^{\text{A}}_{j,+}\begin{pmatrix}1\\ \Lambda_{j,+}\end{pmatrix}e^{ib_{j,+}\xi} + \phi^{\text{A}}_{j,-}\begin{pmatrix}1\\ \Lambda_{j,-}\end{pmatrix}e^{ib_{j,-}\xi}\right], \tag{S5}$$

where C_j is a normalization factor. This can be written as $\Psi_j(\xi) = \Omega_j(\xi)(\alpha_j, \beta_j)^\intercal$ where

$$\Omega_j(\xi) = \begin{pmatrix} e^{ib_{j,+}\xi} & e^{ib_{j,-}\xi} \\ \Lambda_{j,+}e^{ib_{j,+}\xi} & \Lambda_{j,-}e^{ib_{j,-}\xi}\end{pmatrix}, \tag{S6}$$

where $\alpha_j = C_j\phi^{\text{A}}_{j,+}$ and $\beta_j = C_j\phi^{\text{A}}_{j,-}$.

Transfer Matrix

We now consider a smooth waveguide approximated by N square wells as sketched in Fig. S1a. The analytics will be limited to a simple waveguide that terminates at the same maximum potential to the left and right, i.e., $V_1 = V_N = V_{\text{max}}$, but the analysis can be readily extended to the general case. We must first consider our conditions for a bound state. From the perspective of single wells, our bound state must have a plane-wave solution in the deepest well, V_{min}, which means that in this region, $\text{Im}(b_{j,\pm}) = 0$. Additionally, we must ensure that the wavefunction decays in the leftmost and rightmost wells, V_{max}, meaning $\text{Im}(b_{1,\pm}) = \text{Im}(b_{N,\pm}) \neq 0$ so that it disappears as $\xi \to \pm\infty$. These two criteria are satisfied by the condition

$$\{E < V_{\text{min}} + f_1\Delta + f_2|\Delta| \,\cup\, E > V_{\text{min}} + f_1\Delta - f_2|\Delta|\} \,\cap\, \{E > V_{\text{max}} + f_1\Delta + f_2|\Delta| \,\cap\, E < V_{\text{max}} + f_1\Delta - f_2|\Delta|\}, \tag{S7}$$

where

$$f_1 = \frac{st\cos\theta}{l^2}, \tag{S8}$$

and

$$f_2 = -\frac{|T|\sqrt{l^2 - t^2 \sin^2\theta}}{l^2}. \tag{S9}$$

For the reader's convenience these regions have been sketched for two waveguide orientations (see Fig. S1b and c).

Now that we have established the requirements for bound states in the waveguide, we need to match the wavefunctions of each square well at their boundaries. If we begin on the right side of the waveguide, we must match the wavefunctions $\Psi_j(\xi)$ in regions $j = N-1$ and $j = N$ at the interface ξ_{N-1}. This will allow us to write the wavefunction components of region N as a function of the components in region $N-1$:

$$\begin{pmatrix} \alpha_N \\ \beta_N \end{pmatrix} = \Omega_N^{-1}(\xi_{N-1})\Omega_{N-1}(\xi_{N-1}) \begin{pmatrix} \alpha_{N-1} \\ \beta_{N-1} \end{pmatrix}. \tag{S10}$$

Iterating this process will allow us to write the components of the wavefunctions in the right region ($j = N$) as a function of the components of the left region ($j = 1$) as $(\alpha_1, \beta_1)^\intercal = \mathbf{T}(\alpha_N, \beta_N)^\intercal$, where the transfer matrix is defined as

$$\mathbf{T} = \begin{pmatrix} T_{\alpha\alpha} & T_{\alpha\beta} \\ T_{\beta\alpha} & T_{\beta\beta} \end{pmatrix} = \prod_{j=1}^{N-1} \Omega_j^{-1}(\xi_j)\Omega_{j+1}(\xi_j). \tag{S11}$$

To ensure the wavefunction decays at $\xi \to \pm\infty$ we set $\alpha_1 = 0$ and $\beta_N = 0$, hence

$$\begin{pmatrix} 0 \\ \beta_1 \end{pmatrix} = \begin{pmatrix} T_{\alpha\alpha} & T_{\alpha\beta} \\ T_{\beta\alpha} & T_{\beta\beta} \end{pmatrix} \begin{pmatrix} \alpha_N \\ 0 \end{pmatrix}. \tag{S12}$$

This places a constraint on the transfer matrix $T_{\alpha\alpha} = 0$. Therefore, bound states can be found as energies E and wavevectors Δ that satisfy the criteria defined in Eq. (S7) and that ensure $T_{\alpha\alpha} = 0$, which can be found numerically.

References

1. Pereira, J. M., Mlinar, V., Peeters, F. M. & Vasilopoulos, P. Confined states and direction-dependent transmission in graphene quantum wells. *Phys. Rev. B* **74**, 045424, DOI: 10.1103/PhysRevB.74.045424 (2006).
